# Antimicrobial Susceptibility of *Escherichia coli* and ESBL-Producing *Escherichia coli* Diffusion in Conventional, Organic and Antibiotic-Free Meat Chickens at Slaughter

**DOI:** 10.3390/ani10071215

**Published:** 2020-07-17

**Authors:** Laura Musa, Patrizia Casagrande Proietti, Raffaella Branciari, Laura Menchetti, Sara Bellucci, David Ranucci, Maria Luisa Marenzoni, Maria Pia Franciosini

**Affiliations:** Department of Veterinary Medicine, University of Perugia, 06126 Perugia, Italy; laura.musa94@hotmail.com (L.M.); raffaella.branciari@unipg.it (R.B.); laura.menchetti7@gmail.com (L.M.); sarabellucci1988@yahoo.it (S.B.); david.ranucci@unipg.it (D.R.); marialuisa.marenzoni@unipg.it (M.L.M.); maria.franciosini@unipg.it (M.P.F.)

**Keywords:** *E. coli*, ESBL *E. coli*, multi-resistance, meat chickens, rearing system, slaughterhouse

## Abstract

**Simple Summary:**

Following the spread of antibiotic resistance and the high consumption of chicken meat, conventional poultry-producing companies have turned to antibiotic-free and organic lines of products. Our work investigated *E. coli* susceptibility to different antimicrobials and extended-spectrum β-lactamase (ESBL) *E. coli* diffusion from samples collected in slaughterhouse from conventional (C), organic (O) and reared without antibiotics (ABF) chickens. Conventional samples showed the highest number of *E. coli* strains resistant to ampicillin (89.6%), trimethoprim/sulfamethoxazole (62.2%), nalidixic acid (57.8%), ciprofloxacin (44.4%), and cefotaxime (43.7%), with prevalent patterns of multi-resistance to three (35.1%) and to four antimicrobials (31.3%). The highest numbers of ESBL *E. coli* were observed in conventional and the lowest in organic. Our results are relevant with an influence of farming typology regarding the susceptibility of *E. coli* and the presence of ESBL *E. coli*. Conventional farms, in which the use of antibiotics is allowed, showed samples with the highest number of strains resistant to antimicrobials commonly used in poultry as well as the highest amounts of ESBL *E. coli*. Organic samples exhibited the lowest value for ESBL due to a lack of antimicrobial treatment in chickens and the possibility to have access to the outdoors, limiting contact with litter as a potential source of resistant bacteria.

**Abstract:**

As a result of public health concerns regarding antimicrobial resistance in animal-based food products, conventional poultry companies have turned to ‘raised without antibiotics’ (ABF) and organic farming systems. In this work, we evaluated the influence of rearing systems on antimicrobial susceptibility in *E. coli* and extended-spectrum β-lactamase (ESLB) *E. coli* diffusion in conventional (C), organic (O) and antibiotic free (ABF) chicken samples collected from cloacal swabs and skin samples in slaughterhouse. The *E. coli* isolates from conventional (135), antibiotic-free (131) and organic (140) samples were submitted to the Kirby–Bauer method and ESBL *E. coli* were analyzed by the microdilution test. Conventional samples showed the highest number of strains resistant to ampicillin (89.6%; *p* < 0.01), cefotaxime (43.7%; *p* < 0.01), nalidixic acid (57.8%; *p* < 0.01), ciprofloxacin (44.4%; *p* < 0.001), and trimethoprim/sulfamethoxazole (62.2%; *p* < 0.01), with patterns of multi-resistance to three (35.1%) and to four antimicrobials (31.3%), whereas most of the *E. coli* isolated from antibiotic-free and organic chicken samples revealed a co-resistance pattern (29.2% and 39%, respectively). The highest number of ESBL *E. coli* was observed in conventional, in both cloacal and skin samples and the lowest in organic (*p* < 0.001). Our results are consistent with the effect of conventional farming practices on *E. coli* antimicrobial resistance and ESBL *E. coli* number, due to the use of antimicrobials and close contact with litter for most of the production cycle.

## 1. Introduction

The indiscriminate use of antibiotics in poultry has contributed to a progressive increase in bacterial resistance to the main classes of antibiotics such as quinolones, tetracyclines and beta-lactams [1,2]. In addition, the continued exposure of bacterial strains to a large variety of β-lactams has produced the mutation of bacterial β-lactamases, expanding their activity against the newly developed β-lactam antibiotics. These enzymes are known as extended-spectrum β-lactamases (ESBLs). *E. coli* is known to be one of the bacterial species in which the selection of resistance genes has occurred more rapidly over the years following the widespread use of antimicrobials [3]. The genes responsible for resistance are frequently localized in transferable genetic elements such as plasmids and integrons [4,5,6,7], and pathogen and commensal *E. coli* can easily receive antimicrobial resistance genes and transmit them to other bacteria of the intestinal microbiota also by conjugation [8,9,10]. In this scenario, commercial chickens and turkeys are considered an important reservoir of *E. coli* and ESBL multiresistant isolates for humans [11,12]. Vertical transmission of ESBL/*E. coli* through the poultry production pyramid from breeder downward is likely responsible for the origin of bacteria colonizing progeny [13,14,15]. In order to satisfy the health food demand of consumers, most large poultry-producing companies have turned to antibiotic free (ABF) and organic lines of products obtained without the use of antibiotics [16]. Instead of antibiotics, the control of poultry diseases, especially for *E. coli*, relies on the use of autogenous vaccines [17] and on the quality of the environment which the typology of farms is focused [18,19]. Although previous studies reported that the rearing systems, such as antibiotic free or organic farming, play an important role in controlling antibiotic-resistant bacteria in chicken carcasses [20,21], several contradictory aspects are present. Kim et al. [22] reported that *Enterococcus* spp. contamination rates were lower in organic chicken carcasses than in conventional chicken carcasses, as well as the level of resistance to certain antibiotics and the occurrence of multidrug resistance. Oppositely, Parker et al. [23] showed that antibiotic-free retail chicken meat products were also largely contaminated with ESBL-producing Salmonellae and there were not differences between their ESBL genes and those isolated from conventional retail chicken meat products. This work aimed to evaluate the antimicrobial susceptibility of *E. coli* strains and ESLB *E. coli* diffusion, isolated in slaughterhouses from conventional, organic and antibiotic-free chickens.

## 2. Materials and Methods

### 2.1. Sampling

A total of 406 *E. coli* strains were isolated from cloacal swabs and skin samples in slaughterhouse. In particular, 135 *E. coli* were collected from conventional (C) chickens (68 strains from cloacal swabs and 67 from skin samples), 131 from antibiotic-free/raised without antibiotic (ABF) chickens (64 strains from cloacal swabs and 67 from skin samples), and 140 from organic (O) chickens (70 strains from cloacal swabs and 70 from skin samples). Cloacal swabs from 7 subjects for each farm were individually collected at the arrival in the slaughterhouse. Furthermore, for each farm, neck skin fragments (weight of 10 g) were aseptically removed, using sterile scalpels and callipers, from 7 animals at the end of the slaughtering line after chilling. The skin fragments were transferred to sterile bags and stored at refrigeration temperature. To minimize cross-contamination of samples, subjects belonging to the selected farms were slaughtered at the beginning of the slaughtering day. Ten farms for each typology of rearing were investigated. Conventional and (ABF) rearing systems are both indoor and provide the same type of management except for the use of antimicrobials, not provided for ABF, which is used only in the case of bacterial diseases that could compromise the state of well-being of the birds.

### 2.2. Isolation and Identification of E. coli

Both cloacal swabs and skin samples were placed in pre-enrichment medium consisting of buffered peptone water (BPW) in a ratio of 1:10 and were then incubated at 37 °C for 18–24 h in aerobiosis; 0.1 mL from each diluted sample was plated on MacConkey agar and on MacConkey agar added with low concentration (1 mg/L) of cefotaxime (Thermo Fisher Scientific, Rodano, Italy). The plates were incubated for 24 h at 37 °C under aerobic conditions. All colonies with typical *E. coli* morphology were selected and confirmed by biochemical tests (ISO) [24].

### 2.3. Antibiotic Susceptibility Testing and ESBL Detection

To assess the antimicrobial susceptibility, all *E. coli* isolates were analyzed on Mueller-Hinton agar plates (Thermo Fisher Scientific, Rodano, Italy), containing ampicillin (AMP) (10 μg), cefotaxime (CTX) (30 μg), ceftazidime (CAZ) (30 μg), amoxicillin/acid clavulanic (AMC) (30 μg), nalidixic acid (NA) (30 μg), ciprofloxacin (CIP) (5 μg), trimethoprim/sulfamethoxazole (SXT) (25 μg), (TET) tetracycline (30 μg), gentamicin (CN) (10 μg). The plates were incubated at 37 °C for 24 h under aerobic conditions. For all *E. coli*, ESBL production was confirmed by the combined disk test with cefotaxime and ceftazidime alone and in combination with clavulanic acid and by the microdilution method using Sensititre™ extended spectrum beta-lactamase plates (Thermo Fisher Scientific, Rodano, Italy), according to the Clinical and Laboratory Standards Institute (CLSI) guidelines [25].

### 2.4. Statistical Analysis

Generalized linear models (GLMs) were used to evaluate the effect of farm (three levels: conventional, antibiotic-free and organic farms) and sample type (two levels: skin and cloacal swabs). Multinomial and cumulative logit were the probability distribution and the link function, respectively, used to evaluate each antibiotic resistance categorized as sensitive, intermediate and resistant. To analyse ESLB *E. coli*, binomial and logit were used as the probability distribution and the link function, respectively. In addition, z-tests with the Bonferroni correction were used to compare column proportions. To evaluate the factors affecting the number of resistance strains, Poisson distribution and Log link function were used. The multi-resistance patterns including ≥ 10 events per variable (EPV) [26] were coded 0 (negative) and 1 (positive) and analysed by generalized linear models (GLMs). These models used binomial and logit as the probability distribution and the link function, respectively, and evaluated the effect of farm and sample type. Distributions within categorical variables were evaluated using chi-squared goodness of fit tests assuming all categories equal. A *p*-value < 0.05 was considered statistically significant. All analyses were performed using SPSS version 25.0 statistical analysis software (IBM Inc., Chicago, IL, USA).

## 3. Results

**Figure 1:**
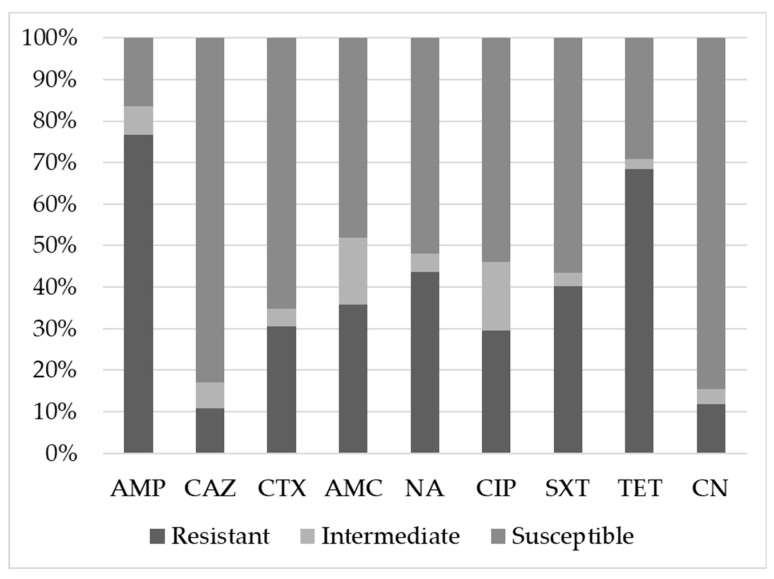
Susceptibility of *E. coli* strains to different antimicrobials. Ampicillin (AMP), cefotaxime (CTX), ceftazidime (CAZ), amoxicillin/acid clavulanic (AMC), nalidixic acid (NA), ciprofloxacin (CIP), trimethoprim/sulfamethoxazole (SXT), tetracycline (TET) and gentamicin (CN).

Regardless of the rearing system and the type of sample, the highest number of resistant *E. coli* was observed for ampicillin (76.6%), followed by tetracycline (68.4%), nalidixic acid (43.6%), trimethoprim/sulfamethoxazole (40.1%), and amoxicillin/acid clavulanic (35.7%). In total, 30.5% and 29.6% of strains were resistant to cefotaxime and ciprofloxacin, respectively, and 11.8% and 10.8% of strains were resistant to gentamicin and ceftazidime, respectively (Figure 1).

Comparing isolate susceptibility on the basis of rearing system (Table 1), the highest levels of resistance for ampicillin (89.6%; *p* = 0.002), cefotaxime (43.7%; *p* = 0.001), nalidixic acid (57.8%; *p* = 0.001), ciprofloxacin (44.4%; *p* < 0.001) and trimethoprim/sulfamethoxazole (62.2%; *p* > 0.001) were observed in *E. coli* isolated from conventional chicken samples. A high prevalence of resistant *E. coli* strains was found in antibiotic-free, organic and conventional samples for tetracycline (71%, 65.7% and 68.9%, respectively). With respect to the type samples, differences in antimicrobial resistance were found for amoxicillin/acid clavulanic and ciprofloxacin (*p* < 0.01; Table 1). In particular, *E. coli* isolated from cloacal samples showed higher proportions of susceptible strains for amoxicillin/acid clavulanic (56.4%) than skin samples (39.6%) (Table S1).

Most *E. coli* isolated from conventional chickens showed a pattern of multi-resistance to three antimicrobials (35.1%), with prevalence of a beta-lactams/trimethoprim-sulfamethoxazole/tetracycline profile (*p* < 0.001) and to four antimicrobials (31.3%) with prevalence of a beta-lactams/quinolones/trimethoprim-sulfamethoxazole/tetracycline profile (*p* < 0.001; Table 2). *E. coli* isolated from antibiotic-free and organic chicken samples revealed a co-resistance pattern of 29.2% and 39.0%, respectively, with prevalence of a beta-lactams/tetracycline profile (*p* < 0.001). A pattern of multi-resistance to five antimicrobials beta-lactams/quinolones/trimethoprim-sulfamethoxazole/tetracycline/gentamicin was found in *E. coli* isolated from conventional (8.4%), antibiotic-free (4.2%) and organic (2.9%) samples (Table 2).

The proportion of *E. coli* resistant to beta-lactams/tetracycline isolated from conventional chickens was lower than *E. coli* isolated from organic and antibiotic-free chickens (*p* < 0.05), but *E. coli* isolated from conventional chickens showed a higher prevalence of multi-resistance toward beta-lactams/quinolones/trimethoprim-sulfamethoxazole/tetracycline than *E. coli* isolated from organic and antibiotic-free farms (*p* < 0.05; Table 3). 

Seventy-two ESBL *E. coli* (18.6%) strains were isolated from all samples; in particular, 19 (27.9%) were isolated from conventional, 7 (10.9%) from antibiotic-free, and 9 (12.9%) from organic cloacal swabs. ESBL *E. coli* isolates from skin samples were 1 from organic (1.4%), 15 from antibiotic-free (22.4%) and 21 from conventional (31.3%) skin samples (*p* < 0.001; Table 4). 

## 4. Discussion

The emergence of the antibiotic resistance in animals and in humans has led to changes in the zootechnical sector aimed at an increasingly progressive reduction in the use of antimicrobials, both as metaphylaxis and therapeutic tools. Farms with management providing for the use of antibiotics only if strictly necessary, such as organic and antibiotic-free farms, have increased, as well as the use of prebiotics or probiotics and autogenous vaccines [17] as alternatives to antimicrobials. From 2017, a National Action Plan on Antimicrobial Resistance (PNCAR) 2017–2020 was adopted in Italy to face the increase in antimicrobial resistance, through the synergy between national, regional and local levels. The most important goals for the veterinary sector have been to reduce the consumption of antibiotics by more than 30% and to reduce the consumption of Critically Important Antimicrobials by more than 10%, in particular the decrease in colistin consumption to a level of 5 mg/PCU (Population Correction Units) [27]. In our work, independent of the typology of sampling and rearing system, the highest number of *E. coli* resistant strains were found for ampicillin, tetracycline and nalidixic acid, followed by trimethoprim/sulfamethoxazole and amoxicillin/acid clavulanic. All these molecules have been used in poultry therapy and prevention over the years, favouring the selection of resistant bacteria [28] that are shareable at the human community via food or environmental contamination, as well as direct contact with animals [29,30,31]. The numbers of *E. coli* strains resistant to ampicillin, nalidixic acid, cefotaxime, ciprofloxacin, trimethoprim/sulfamethoxazole were significantly higher in conventional chicken samples, supporting the hypothesis that the use of antibiotics in this management system can provide selective pressure on the microbial community, thereby facilitating the persistence and transfer of resistance determinants among bacterial species [32,33]. A recent study showed a higher prevalence of resistance among *E. coli* isolates from conventional meat turkeys in comparison to antibiotic-free and organic turkeys but no differences in antimicrobial susceptibility were seen in *E. coli* isolates obtained from meat chickens [34]. Mollenkopf et al. [35] did not find any variation in bacteria resistant to critically important antimicrobial drugs, such as cephalosporins, polymyxins, quinolones and macrolides [36] among organic, antibiotic-free and conventional retail chicken products. In our work, a number *of E. coli* strains resistant to tetracycline and ampicillin were also isolated from antibiotic-free and organic samples in spite of the absence of antimicrobial use. The action exerted by the outdoor, often contaminated by resistant bacteria from soil or spread via wild birds, should not be neglected in organic system farming [37,38,39]. The presence of resistant strains in day-old chicks, probably infected by the vertical route, should be also mentioned [40,41]. It has been hypothesized that contamination occurs in the hatchery itself or during transport to farm [42,43]; at this moment, early colonization of resistant bacteria could be also favoured by the presence of immature and not sufficiently competitive intestinal flora [44]. Samplings seemed to influence significantly the number of *E. coli* resistant to ciprofloxacin in conventional chicken and the number of *E, coli* susceptible to amoxicillin/clavulanic acid in organic chicken samples. Considering the typology of samples, in our work, *E. coli* isolated from cloacal samples showed higher proportions of susceptible strains for amoxicillin/acid clavulanic than *E. coli* isolated in skin samples. In this context, a short fasting period for meat chicken before transport to the slaughterhouse in order to reduce stool release could play a role. There is no doubt that the slaughterhouse represents an environment responsible for cross-contamination by resistant bacteria, especially during the scalding, killing and evisceration of poultry [45]. Staff, air pollution and equipment can also contribute to the persistence of contamination in the clean area. In relation to multi-resistance, most *E. coli* isolated from conventional chicken samples were resistant to three or four antimicrobials, while the co-resistance was more frequently seen in organic and antibiotic-free. The prevalence of a resistance pattern to four antibiotics was higher in conventional than organic and antibiotic-free samples. The multi-resistance profiles were frequently represented by tetracycline, trimethoprim/sulfamethoxazole and molecules included in the beta-lactams and quinolones classes, due to their extensive use in poultry farming over the years. It is known that multi-drug resistance is common in poultry [46,47] and *E. coli* from industrial broilers show resistance to multiple antibacterial agents used in human therapy [48,49,50]. Moreover, the degree of multi-resistance in *E. coli* isolates is highest in broiler chickens in comparison to other livestock groups [48]. Chuppava et al. [51] showed that close contact with litter for most of the production cycle can influence the presence of multi-resistant strains in conventionally reared chickens. Our findings agree with previous investigations reporting resistance in *E. coli* isolated from conventional poultry farms to many classes of antibiotics, including fluoroquinolones β-lactams, tetracyclines and sulphonamides [47,49,51]. Conventional chickens showed the highest percentage of ESBL *E. coli*, in cloacal and skin samples, followed by organic chickens, while organic chickens exhibited the lowest percentage in skin samples. The number of ESBL E. coli seemed to be affected by the rearing system since the lowest value for organic chicken samples could support the hypothesis that they have access to the outdoors and contact with litter was more limited in comparison to conventional and antibiotic-free chickens reared exclusively indoor. Our results disagree with previous investigations performed on both organic and conventional meat chicken products reporting that there were no considerable differences in ESBL producing *E. coli*, ESBL genes and strain types [52]. In recent years, ESBL bacteria from food animal products have represented an alarming threat to public health, since beta-lactams are commonly used in humans, alone or associated with beta-lactamase inhibitors. A recent study reported that cephalosporin-resistant *E. coli* clones detected in humans are frequently associated with human disease and originate from food animal products [53], although other studies have demonstrated that the contribution from poultry is limited [54,55]. 

## 5. Conclusions

We conclude that the typology of rearing systems is associated with antibiotic resistance since conventional samples showed the highest numbers of *E. coli* strains resistant to the most of antimicrobials tested as well as the strains with multi-resistant profiles. A limited contact with the litter during their productive life could justify the low count of ESBL *E. coli* reported in organic samples in comparison to other systems However, it should not be overlooked that a number of resistant *E. coli* strains were recovered from both organic and antibiotic-free samples. Vertical transmission and/or early contamination at hatch should be considered in these farming typologies. Furthermore, external environment contamination could play a role on the presence of resistant bacteria in organic farm. Sampling seemed to have a significant effect on the recovery of the number of resistant strains for ciprofloxacin in conventional farm, but it plays a less substantial role than the typology of rearing systems. Further studies are necessary to deepen our knowledge of the epidemiological circuit of antibiotic resistance, and frequent and continuous monitoring of the chicken production chain should be performed in order to optimize biosecurity measures and ensure final product safety.

## Figures and Tables

**Table 1:** Effect of sampling and different rearing systems on susceptibility of *E. coli* isolates.

Antimicrobials		Farm						*p* Value		
		ABF		O		C		Farms	Samples	Interaction
		*E. coli* (No)	%	*E. coli* (No)	%	*E. coli* (No)	%			
**AMP**	Resistant	89 ^a^	67.9%	101 ^a^	72.1%	121 ^b^	89.6%	**0.002**	0.154	0.051
	Intermediate	13 ^a^	9.9%	12 ^a,b^	8.6%	3 ^b^	2.2%			
	Susceptible	29 ^a^	22.1%	27 ^a^	19.3%	11 ^b^	8.1%			
**CAZ**	Resistant	12 ^a,b^	9.2%	8 ^b^	5.7%	24 ^a^	17.8%	**0.001**	0.791	0.230
	Intermediate	13 ^a^	9.9%	2 ^b^	1.4%	10 ^a^	7.4%			
	Susceptible	106 ^a^	80.9%	130 ^b^	92.9%	101 ^a^	74.8%			
**CTX**	Resistant	31 ^a^	23.7%	34 ^a^	24.3%	59 ^b^	43.7%	**0.001**	0.520	**0.003**
	Intermediate	8 ^a^	6.1%	4 ^a^	2.9%	5 ^a^	3.7%			
	Susceptible	92 ^a^	70.2%	102 ^a^	72.9%	71 ^b^	52.6%			
**AMC**	Resistant	44 ^a^	33.6%	44 ^a^	31.4%	57 ^a^	42.2%	0.114	**0.002**	**0.016**
	Intermediate	26 ^a^	19.8%	16 ^a^	11.4%	24 ^a^	17.8%			
	Susceptible	61 ^a^	46.6%	80 ^b^	57.1%	54 ^a^	40.0%			
**NA**	Resistant	44 ^a^	33.6%	55 ^a^	39.3%	78 ^b^	57.8%	**0.001**	0.683	0.087
	Intermediate	12 ^a^	9.2%	2 ^b^	1.4%	4 ^a,b^	3.0%			
	Susceptible	75 ^a^	57.3%	83 ^a^	59.3%	53 ^b^	39.3%			
**CIP**	Resistant	27 ^a^	20.6%	33 ^a^	23.6%	60 ^b^	44.4%	**<0.001**	**0.005**	**0.008**
	Intermediate	15 ^a^	11.5%	24 ^a^	17.1%	28 ^a^	20.7%			
	Susceptible	89 ^a^	67.9%	83 ^a^	59.3%	47 ^b^	34.8%			
**SXT**	Resistant	44 ^a^	33.6%	35 ^a^	25.0%	84 ^b^	62.2%	**<0.001**	0.952	**0.038**
	Intermediate	8 ^a^	6.1%	4 ^a, b^	2.9%	1 ^b^	0.7%			
	Susceptible	79 ^a^	60.3%	101 ^a^	72.1%	50 ^b^	37.0%			
**TET**	Resistant	93 ^a^	71.0%	92 ^a^	65.7%	93 ^a^	68.9%	0.645	0.642	0.326
	Intermediate	5 ^a^	3.8%	5 ^a^	3.6%	0 ^a^	0.0%			
	Susceptible	33 ^a^	25.2%	43 ^a^	30.7%	42^a^	31.1%			
**CN**	Resistant	15 ^a, b^	11.5%	10 ^b^	7.1%	23 ^a^	17.0%	0.185	0.775	0.083
	Intermediate	9 ^a^	6.9%	5 ^a, b^	3.6%	1 ^b^	0.7%			
	Susceptible	107 ^a^	81.7%	125 ^a^	89.3%	111 ^a^	82.2%			

^a, b^ Values in the same row followed by the same letter (^a^ or ^b^) do not differ significantly (*p* < 0.05; Z test and Bonferroni correction); antibiotic-free (ABF), organic (O), conventional (C); Ampicillin (AMP), cefotaxime (CTX), ceftazidime (CAZ), amoxicillin/acid clavulanic (AMC), nalidixic acid (NA), ciprofloxacin (CIP), trimethoprim/sulfamethoxazole (SXT), tetracycline (TET) and gentamicin (CN).

**Table 2:** Resistance patterns in *E. coli* isolated from antibiotic free (ABF) organic (O), and conventional (C) samples collected in slaughterhouse.

Pattern	Farm						Antimicrobial Resistance Pattern	Farm					
	ABF		O		C			ABF		O		C	
	*E. coli* (No)	%	*E. coli* (No)	%	*E. coli* (No)	%		*E. coli* (No)	%	*E. coli* (No)	%	*E. coli* (No)	%
1	28	23.3%	37	27.2%	10	7.6%							
2	35	29.2%	53	39.0%	23	17.6%	BL/CN	1	0.3%	0	0.0%	0	0.0%
							BL/QUIN	4	1.0%	13	3.4%	3	0.8%
							BL/SXT	0	0.0%	6	1.6%	12	3.1%
							BL/TET	26	6.7%	25	6.5%	7	1.8%
							QUIN/CN	0	0.0%	1	0.3%	1	0.3%
							QUIN/SXT	0	0.0%	1	0.3%	0	0.0%
							QUIN/TET	2	0.5%	7	1.8%	0	0.0%
							SXT/TET	1	0.3%	0	0.0%	0	0.0%
							TET/CN	1	0.3%	0	0.0%	0	0.0%
3	32	26.7%	23	16.9%	46	35.1%	BL/QUIN/CN	0	0.0%	1	0.3%	0	0.0%
							BLQUIN/SXT	3	0.8%	2	0.5%	11	2.8%
							BL/QUIN/TET	12	3.1%	15	3.9%	20	5.2%
							BL/SXT/TET	14	3.6%	3	0.8%	12	3.1%
							BL/TET/CN	2	0.5%	1	0.3%	2	0.5%
							QUIN/SXT/TET	1	0.3%	1	0.3%	1	0.3%
4	20	16.7%	19	14.0%	41	31.3%	BL/QUIN/SXT/CN	0	0.0%	0	0.0%	2	0.5%
							BL/QUIN/SXT/TET	19	4.9%	17	4.4%	32	8.3%
							BL/QUIN/TET/CN	0	0.0%	2	0.5%	4	01.0%
							BL/SXT/TET/CN	1	0.3%	0	0.0%	3	0.8%
5	5	4.2%	4	2.9%	11	8.4%	BL/QUIN/SXT/TET/CN	5	1.3%	4	1.0%	11	2.8%

BL = beta-lactams (AMP, AMC, CAZ, CTX); QUIN = quinolones (CIP, NA), SXT = trimethoprim/sulfamethoxazole, TET = tetracycline, CN = gentamicin.

**Table 3:** Effects of farm typology and samplings on resistance profiles.

Antimicrobial Resistance Pattern	Farms						*p* Value	
	ABF		O		C		Farm	Sample
	*E. coli* (No)	%	*E. coli* (No)	%	*E. coli* (No)	%		
BL/QUIN	4 ^a,b^	2.9%	13 ^b^	9.3%	3 ^a^	2.1%	**0.012**	1.000
BL/TET	26 ^a^	18.6%	25 ^a^	17.9%	7 ^b^	5.0%	**0.002**	0.587
BL/QUIN/TET	12 ^a^	8.6%	15 ^a^	10.7%	20 ^a^	14.3%	0.287	0.655
BL/SXT/ TET	14 ^a^	10.0%	3 ^b^	2.1%	12 ^a,b^	8.6%	**0.041**	0.835
BL/QUIN/SXT/TET	19 ^a^	13.6%	17 ^a^	12.1%	32 ^b^	22.9%	**0.041**	0.165
BL/QUIN/SXT/TET/CN	5 ^a^	3.6%	4 ^a^	2.9%	11 ^a^	7.9%	0.130	0.349

^a, b^ Values in the same row followed by the same letter (^a^ or ^b^) do not differ significantly (*p* < 0.05); BL = beta-lactams (AMP, AMC, CAZ, CTX), QUIN = quinolones (CIP, NA), SXT = trimethoprim/sulfamethoxazole, TET = tetracycline, CN = gentamicin; antibiotic-free (ABF), organic (O), conventional (C).

**Table 4:** ESBL producing *E. coli* isolates in cloacal and skin samples from antibiotic-free (ABF), organic (O) and conventional (C) chickens.

Sample Type		Farm Type						*p* Value		
		ABF		O		C		Farm	Sample	Interaction
		ESBL *E. coli* (No)	%	ESBL *E. coli* (No)	%	ESBL *E. coli* (No)	%			
**Cloacal**	Negative	57 ^a^	89.1%	61 ^a^	87.1%	49 ^b^	72.1%	**<0.001**	0.281	**0.021**
	Positive	7 ^a^	10.9%	9 ^a^	12.9%	19 ^b^	27.9%			
**Skin**	Negative	52 ^a^	77.6%	69 ^b^	98.6%	46 ^a^	68.7%			
	Positive	15 ^a^	22.4%	1 ^b^	1.4%	21 ^a^	31.3%			

Values in the same row followed by the same letter do not differ significantly (*p* < 0.05; Z test and Bonferroni correction.

## Supplementary Materials

The following are available online at https://www.mdpi.com/2076-2615/10/7/1215/s1, Table S1. Susceptibility of *E. coli* isolated in cloacal and skin sample.
