# Supplementary material for: Antimicrobial Susceptibility of Escherichia coli and ESBL-Producing Escherichia coli Diffusion in Conventional, Organic and Antibiotic-Free Meat Chickens at Slaughter
Source: Animals (Basel). 2020 Jul 17;10(7):1215. doi: 10.3390/ani10071215 (PMC7401526; doi:10.3390/ani10071215)
Supplement: Supplementary file 1 [file animals-10-01215-s001.pdf]

Table S1. Susceptibility of *E. coli* isolated in cloacal and skin sample.

		Sample			
		**Skin**		**Cloacal swabs**	
		**Count**	**%**	**Count**	**%**
**AMP**	Resistant	158a	78.2%	153a	75.0%
	Intermediate	15a	7.4%	13a	6.4%
	Susceptible	29a	14.4%	38a	18.6%
**CAZ**	Resistant	22a	10.9%	22a	10.8%
	Intermediate	10a	5.0%	15a	7.4%
	Susceptible	170a	84.2%	167a	81.9%
**CTX**	Resistant	62a	30.7%	62a	30.4%
	Intermediate	4a	2.0%	13b	6.4%
	Susceptible	136a	67.3%	129a	63.2%
**AMC**	Resistant	74a	36.6%	71a	34.8%
	Intermediate	48a	23.8%	18b	8.8%
	Susceptible	80a	39.6%	115b	56.4%
**NA**	Resistant	83a	41.1%	94a	46.1%
	Intermediate	15a	7.4%	3b	1.5%
	Susceptible	104a	51.5%	107a	52.5%
**CIP**	Resistant	52a	25.7%	68a	33.3%
	Intermediate	26a	12.9%	41b	20.1%
	Susceptible	124a	61.4%	95a	46.6%
**SXT**	Resistant	83a	41.1%	80a	39.2%
	Intermediate	5a	2.5%	8a	3.9%
	Susceptible	114a	56.4%	116a	56.9%
**TET**	Resistant	136a	67.3%	142a	69.6%
	Intermediate	6a	3.0%	4a	2.0%
	Susceptible	60a	29.7%	58a	28.4%
**CN**	Resistant	22a	10.9%	26a	12.7%
	Intermediate	7a	3.5%	8a	3.9%
	Susceptible	173a	85.6%	170a	83.3%

Ampicillin (AMP), cefotaxime (CTX), ceftazidime (CAZ), amoxicillin/acid clavulanic (AMC), nalidixic acid (NA), ciprofloxacin (CIP), trimethoprim/sulfamethoxazole (SXT), tetracycline (TET) and gentamicin (CN)
